# A Call for the Inclusion of Mixed Methods Research in the Undergraduate Psychology Curriculum

**DOI:** 10.3389/fpsyg.2018.02709

**Published:** 2019-01-08

**Authors:** Lynne D. Roberts, Peter J. Allen

**Affiliations:** ^1^School of Psychology, Curtin University, Perth, WA, Australia; ^2^School of Psychological Science, University of Bristol, Bristol, United Kingdom

**Keywords:** mixed methods research, undergraduate psychology, research methods, pedagogy, undergraduate students

For mixed methods research in psychology to expand, a body of psychologists, and psychology academics who have the knowledge and expertise to conduct and review mixed methods research is required. Reviews of mixed methods articles in psychology (e.g., Bartholomew and Brown, 2012; Bartholomew and Lockard, 2018) have highlighted issues related to the lack of clarity of methods used in published mixed methods psychological research. These include the failures to identify the type of mixed method design, the mixed methods research question, the qualitative analysis methodology, and to explicitly state the process for integrating data. These findings highlight the lack of training for psychologists in both conducting and reviewing mixed methods research. Whilst the new American Psychological Association reporting standards for mixed methods research (Levitt et al., 2018) are helpful for authors and reviewers with some mixed methods expertise, they must be complemented by at least foundational training in mixed methods research to ensure the quality and rigor of mixed methods research published in psychology journals.

In order to develop a body of psychologists and psychology academics who have the knowledge and expertise to conduct and review mixed methods studies, we need to teach current and future generations of psychology students about mixed methods research. At present, mixed methods research training, where offered at all, is typically provided at the doctoral level and across disciplines (Christ, 2009; Baran, 2010; Poth, 2014). Whilst Onwuegbuzie et al. (2011) argued that we can expect mixed methods to be routinely taught in the majority of higher degree by research programs in the future, Poth (2014) viewed one-off mixed methods courses as inadequate preparation, calling instead for the teaching of mixed methods research to begin earlier, positioned alongside the teaching of qualitative and quantitative research. Similarly, while not specific to psychology, the Mixed Methods Task Force report on the future of mixed methods (Mertens et al., 2016a) has recommended the inclusion of mixed methods approaches in the undergraduate curriculum, describing this as “the natural starting point” (p. 19).

We echo the sentiment of the Task Force by calling for the inclusion of mixed methods research in the undergraduate psychology curriculum, beginning with teaching an appreciation of mixed methods research. The last decade has seen an increase in the number of psychology undergraduate programs in the United Kingdom, United States of America and Canada^1^ teaching both quantitative and qualitative research methods (Gibson and Sullivan, 2018; McMullen and Winston-Proctor, 2018). Although these methods are often poorly integrated, and many educators tend to focus excessively on differences between qualitative and quantitative paradigms (Fielden et al., 2012), this teaching does provide a foundation for the introduction of mixed methods research. As exposure to quantitative and qualitative research increases, psychology students' perceptions of the incompatibility of qualitative and quantitative research methods decrease (Roberts and Povee, 2014). While the teaching of both quantitative and qualitative research methods equips students for multimethods research (the use of multiple methodologies to address different goals within a research project), mixed methods research varies from multimethods research in the focus on integration, as both qualitative and quantitative components are addressing the same objective (Anguera et al., 2018). Integration can occur during design, methods, interpretation or reporting (Fetters et al., 2013) and can result in additional insights and understanding. When moving from teaching qualitative and quantitative research methods separately to teaching mixed methods research, it is this mixing and integration that needs to be taught. Previous research has demonstrated that undergraduate psychology students are receptive to mixed methods, despite some misconceptions about mixed methods research and skepticism about the motivation and practices of mixed methods researchers (Povee and Roberts, 2015) that may stem from their unfamiliarity with this type of research.

Burgeoning literature on mixed methods research pedagogy at the postgraduate level (e.g., Earley, 2007; Christ, 2009; Baran, 2010; Onwuegbuzie et al., 2011; Frels et al., 2012, 2014; Poth, 2014) can be used to inform the development of mixed methods research courses at the undergraduate level. Frels et al. (2014) reported that leading mixed methodologists emphasized application and integration as learning goals when teaching mixed methods research. However, there is no “one size fits all” in terms of how mixed methods training can be delivered. Research conducted with experienced mixed methods teachers (Onwuegbuzie et al., 2011) indicates that courses vary in their orientation (importance placed on coverage of quantitative and qualitative methodologies before covering mixed methods), application (from conceptual to applied), and structure (from highly structured to exploratory/experiential), highlighting the possible range of teacher positions for mixed methods courses. This diversity of teaching approaches reflects the diversity of mixed methods research designs. Regardless of the teaching approach selected, Mertens et al. (2016a) highlight the role of educators to support students in aligning mixed methods research choices with philosophical assumptions.

A limited number of example models (e.g., Onwuegbuzie et al., 2013; Ivankova and Plano Clark, 2018) and syllabi (e.g., Earley, 2007; Christ, 2009) for mixed methods research courses are available. We encourage academics who teach mixed methods to undergraduates to share their syllabi, resources and experiences. We note that there are currently no materials that relate to teaching mixed methods research in the “statistics and research methods” teaching resources and “research methods” project syllabi sections of the *Society for the Teaching of Psychology* website (http://teachpsych.org/)^2^. The recently published *Oxford Handbook of Undergraduate Psychology Education* (Dunn, 2015) is similarly silent with regard to teaching mixed methods.

Whilst the availability of teaching resources, example syllabi and authoritative guides will no doubt encourage some instructors to introduce mixed methods into undergraduate research methods classes, the inclusion of mixed methods in our accreditation guidelines and textbooks will have a much larger and more immediate impact. In Australia, where the first author works, the Australian Psychology Accreditation Council (2018) require that research methods be taught to all undergraduate psychology students, but are not prescriptive about the range of methods students should be exposed to. In the United Kingdom (home of the second author), the British Psychological Society (2017a,b) specify that undergraduates should learn how to conduct quantitative and qualitative research, but make no mention of mixed methods research. The regulatory landscape in the United States is similar (American Psychological Association, 2016). Furthermore, the current^3^ bestselling psychology research methods textbook on Amazon.com (Morling, 2018) does not include any coverage of qualitative, let alone mixed methods research. Coolican (2014), the current best seller on Amazon.co.uk, does provide coverage of both, although mixed methods is relegated to a handful of brief mentions in chapters on measurement and qualitative methods.

The positioning of mixed methods teaching within the undergraduate psychology curriculum needs careful consideration. Ideally, the way we teach research methods will change, integrating the teaching of qualitative and quantitative methods from the beginning, rather than teaching each separately and in opposition to each other (for an illustration of this approach, see Onwuegbuzie et al., 2010). This will overcome two key challenges currently faced in teaching mixed methods: the preconceived bias and misperceptions of students about quantitative and qualitative methods and the diversity of competence in qualitative and quantitative research of students entering postgraduate mixed methods research courses (Frels et al., 2012).

Where this restructuring of the research methods curriculum is not possible, there are a number of alternatives. First, it is possible to teach quantitative, qualitative and mixed methods courses sequentially throughout the undergraduate curriculum, such as is currently done at the first author's institution. A potential consequence of this sequential approach is that quantitative research is set up as the main research methodology for psychology, with qualitative research then seen as an alternative approach (Roberts and Castell, 2016). Mertens et al. (2016a) note that where either quantitative or qualitative approaches are priveliged in teaching, a cultural shift will be required to support mixed methods approaches. The priveliging of quantaitive research methods over qualitative research methods in order of teaching potentially leads to a misperception that the qualitative component of mixed methods research is tokenistic (Povee and Roberts, 2015).

Beyond this, decisions on how to incorporate the teaching of mixed methods research in the undergraduate psychology curriculum can be informed by the three components of a student-centered pedagogy for research methods identified by Kilburn et al. (2014): making the research process visible, engaging students in conducting research, and reflecting on the research process. Examples of activities that fit within this framework might include embedding mixed methods assignments and presenting and discussing relevant mixed methods research findings within subject courses. Greene (2010) asks students to select a mixed methods article and lead a class discussion on the quality of the study. The first author has individual and group assessments that require students to take the perspective of a journal article reviewer and critically review mixed methods articles. These tasks explicitly ask students to reflect on how well the qualitative and quantitative components of the research haved been integrated.

Regardless of the way in which mixed methods research is implemented within the undergraduate psychology curriculum, evaluation will be essential in order to determine “what works.” Guetterman et al. (2017) have developed a reliable and validated self-rated measure of mixed methods skills that can be used to track students' self-ratings of skill development, and may be used by course coordinators to improve curricula in areas where students report they have limited skills.

We are not suggesting that introducing mixed methods to the undergraduate psychology curriculum will be an easy task. A key challenge facing mixed methods research educators is the time required to cover key quantitative and qualitative methods in addition to the mixing of methods (Onwuegbuzie et al., 2011; Frels et al., 2012). Students in graduate courses report finding mixed methods terminology and the concept of mixing paradigms challenging (Hesse-Biber, 2015; Gilmartin and Esterhuizen, 2017). This is not surprising given the “kaleidoscope” of mixed methods approaches and terminology (Mertens et al., 2016b) and questions over the relevant content and skills to be taught (Mertens et al., 2016b). Teachers of mixed methods courses often have not been formally taught mixed methods themselves, and may have strengths in only qualitative or quantitative research, indicating that a team-teaching approach may in some cases be required (Hesse-Biber, 2015). Further, in our experience, not all students are interested in learning mixed methods research, with some undergraduate students already expressing a clear preference for either quantitative or qualitative research methodologies by their final undergraduate year. Despite these challenges, we envisage there will be benefits to the discipline and employers in future years in terms of producing psychology graduates who are able to select from the full range of research designs: quantitative, qualitative and mixed methods.

## Author Contributions

LR was responsible for leading the writing of the paper. PA contributed to the design, ideas and writing.

### Conflict of Interest Statement

The authors declare that the research was conducted in the absence of any commercial or financial relationships that could be construed as a potential conflict of interest.

## Footnotes

^1^The teaching of qualitative research methods in undergraduate psychology in other countries has not been documented in the academic literature. We acknowledge that there are likely to be large variations in the amount of qualitative research methods taught across countires, and this will impact on the feasibility of introducing mixed methods research.

^2^We note that the only graduate level research methods project syllabus listed also neglects mixed methods research.

^3^At 28 September 2018.
